# Hemimegalencephaly in Children: A Systematic Review of Diagnosis, Management, and Outcomes in 161 Reported Cases

**DOI:** 10.3390/children13101292

**Published:** 2026-09-23

**Authors:** Farah Mokeddem, Maria Veronica Moran, Alex S. Aguirre, Diego Moreno, Rinat Jonas, Juan Contreras, Yasmine Elhefnawy, Alcy R. Torres

**Affiliations:** 1 Division of Pediatric Neurology, Department of Pediatrics, Boston University Chobanian and Avedisian School of Medicine, Boston, MA 02118, USAmoranpom@nychhc.org (M.V.M.); alex.aguirrebonilla@childrens.harvard.edu (A.S.A.); diego.m@wustl.edu (D.M.); rjonas@bu.edu (R.J.); yelhefn1@bu.edu (Y.E.); 2 Department of Pediatrics, Boston Children’s Hospital, Harvard Medical School, Boston, MA 02115, USA; 3 Pediatric Neurology Department, Universidad Andres Bello, Santiago 8370035, Chile; juan.contreras@edumedica.cl; 4 Pediatric Neurology Department, Universidad de Almeria, 04120 Almeria, Spain; 5 Pediatric Brain Injury Program, Boston Medical Center, 801 Albany Street, Boston, MA 02482, USA

**Keywords:** hemimegalencephaly, children, seizure, treatment, surgery, prognosis

## Abstract

**Highlights:**

**What are the main findings?**

• In our study with 161 cases of pediatric hemimegalencephaly, isolated HME was the most common subtype, seizures typically began before age one (most often focal-onset with impaired awareness), and drug-resistant epilepsy affected 73% of the study sample.
• In terms of neurosurgical procedures, functional procedures were more frequently reported than anatomical and the limited available literature suggests broadly comparable seizure outcomes.
• Genetic testing (positive in only ~9.3% of cases) most often identified PIK3CA and NPRL3 variants, which could correlate with the fact that HME is primarily driven by somatic mosaic mutations in the PI3K–AKT–mTOR pathway, while associated neurocutaneous findings, especially Hypomelanosis of Ito and tuberous sclerosis, frequently served as visible clinical clues to the underlying diagnosis even without molecular confirmation.

**What are the implications of the main findings?**

• Because HME often escapes genetic confirmation (due to mosaicism and limited tissue-specific testing), clinicians should rely on a combined approach both recognizing cutaneous and somatic markers alongside imaging and EEG findings to support earlier diagnosis and guide timely, individualized management.
• Given the high rate of drug-resistant epilepsy and the comparable efficacy of functional versus anatomical hemispherectomy, early referral for multidisciplinary evaluation (neurology, genetics, radiology, neurosurgery) is essential to optimize seizure control and developmental outcomes, while larger, longer-term studies are still needed to refine surgical decision-making and explore emerging alternatives like mTOR-targeted therapy.

**Abstract:**

Background/Objectives: To describe the clinical and genetic features of hemimegalencephaly in pediatric patients, and to highlight treatment outcomes in this population. Methods: This is a systematic literature review following PRISMA guidelines. We used PubMed, Google Scholar, and Scopus for this systematic literature review as databases. Clinical data, imaging findings, EEG patterns, genetic variants, associated syndromes and treatment modalities and outcomes were reviewed, analyzed and summarized in tables. A total of 161 pediatric patients with hemimegalencephaly are described. A risk of bias and sensitivity analysis was conducted. Results: Hemimegalencephaly is more frequently reported in males. The most common subtype was isolated hemimegalencephaly followed by the syndromic subtype, in particular the association with Ito’s hypomelanosis. The most common seizure type was focal onset with impaired awareness. Genetic analysis showed variants in TSC1, TSC2, NPRL3, and PIK3CA, supporting mTOR pathway involvement. Drug-resistant epilepsy was present in approximately 73.2% (118/161) of the sample size. Hemispherectomy, especially functional, in our sample was more frequently reported and the current limited available literature suggests potentially comparable seizure outcomes compared to anatomical hemispherectomy in terms of seizure reduction. Our biggest limitation is the lack of long-term neurological follow-up in the existing literature. Conclusions: Hemimegalencephaly presents with a wide clinical and genetic spectrum and frequently results in drug-resistant epilepsy and developmental impairment. Although surgery may offer seizure relief, outcomes vary and the optimal approach remains individualized. Early diagnosis is essential to enable timely intervention and coordinated care. A multidisciplinary team including neurology, genetics, radiology and neurosurgery is key to optimizing outcomes. Broader studies including milder cases and long follow-up are needed to fully understand disease progression and guide management.

## 1. Introduction

Hemimegalencephaly (HME) is a rare congenital brain malformation characterized by abnormal asymmetric neuronal proliferation leading to enlargement of one cerebral hemisphere [1,2,3]. Genetic factors and mutations in the mTOR (mammalian target of rapamycin) pathway are related to the pathogenesis of this disease [4]. Despite growing recognition, HME remains under-diagnosed, and epidemiological data are limited. It is estimated that HME accounts for approximately 1–3 per 1000 children diagnosed with epilepsy [5]. Clinically, HME often presents in early childhood with drug-resistant epilepsy (DRE), developmental delay, hemiparesis, and hemianopia, among other neurological abnormalities [6]. There are three main types of HME: isolated HME, syndromic HME occurring as part of a genetic or neurocutaneous disorder, and total HME which additionally involves the brainstem and cerebellum [4]. Given its early onset and the importance of the first years of life for neurological development, HME can significantly affect cognitive, motor, and social outcomes [5]. Early recognition improves management options, however delayed diagnosis often occurs, due to its rarity and variable presentation. Most patients with HME present with contralateral hemiparesis, cognitive impairment, and DRE often requiring neurosurgery [7]. Usually, medical therapy, diets, and neurostimulation serve only as bridges while awaiting surgery. Currently, the choice between anatomic and functional hemispherectomy approaches are actively debated given their outcomes and complications [7,8]. Consequently, managing HME requires balancing these surgical risks against the goal of halting cognitive decline, while emerging options like investigational transarterial embolization and targeted mTOR pathway inhibitors could be potential alternatives or adjuncts to surgery in the future with pharmacogenomics [9,10]. This systematic literature review summarizes current knowledge on HME in the pediatric population, focusing on clinical features, diagnostic evaluation, imaging characteristics, surgical approaches, and prognosis.

## 2. Materials and Methods

We performed a systematic review using the PRISMA 2020 guideline to gather information about demographics, types of HME, associated syndromes or clinical findings, associated epilepsy or drug-resistant epilepsy, and surgical management of HME. Figure 1 shows the results of the study using this guideline. PRISMA 2020 checklist see Supplementary Martial. The systematic review was not registered before its initiation, and a protocol was not prepared. Gray literature was used for the systematic review to fill knowledge gaps about imaging, EEG findings, and surgical complications not captured in the main systematic review to provide a more robust analysis given the lack of uniform data found in case studies and case reports which are the more prevalent studies reflecting these data.

Drug-resistant epilepsy was defined as failure of adequate trials of two tolerated, appropriately chosen and used antiepileptic drug schedules to achieve sustained seizure freedom as per the International League Against Epilepsy [11]. The proportion of DRE patients in our sample was calculated by identifying patients who met these criteria and were recognized by the authors as such.

To minimize patient overlap across studies and to generate a unique, non-redundant pooled cohort, publication year, patient age, and country of origin were used as the primary criteria for cross-referencing cases. The majority of studies included in this systematic review reported a single patient, typically presenting at a young age (ranging from neonatal to infancy), from geographically distinct countries. Furthermore, the studies contributing larger sample sizes originated from Italy, the United States, Turkey, and Japan, published in different years and involving patients of varying ages. Notably, these larger series report patients treated at the authors’ own institutions, indicating that the reported cases originate from a single center’s local patient population rather than being aggregated from other countries or referral centers abroad. Based on this distribution of publication years, countries, and patient ages, together with the single-center origin of the larger series, we infer that the degree of patient overlap, if present, is likely minimal.

### 2.1. Inclusion Criteria

1. Language: Studies must be written in English.
2. Population: Studies must include pediatric patients.
3. Focus: Studies must report data on HME.
4. Analysis: Studies must contain an analysis of HME and treatment.
5. Outcomes reported:
  - Characteristics of the HME;
  - Treatment outcome;
  - Main conclusions of each study.

Exclusion criteria

1. Linguistic exclusion: Studies not written in English were excluded.
2. Animal studies: Studies involving animals were excluded.
3. Nonrelevant focus: Articles not involving HME were excluded.
4. Population exclusion: Studies not involving pediatric patients were excluded.
5. Irrelevant HME analysis: Studies not containing an analysis of HME and treatment outcome were excluded.

### 2.2. Data Collection

We used PubMed, Google Scholar, and Scopus for this systematic literature review. The search was conducted between 15 December 2025, and 15 July 2026. We used an advanced search strategy with the following terms for Pubmed: (“hemimegalencephaly”[Title/Abstract] AND “pediatric”[Title/Abstract] AND “epilepsy”[Title/Abstract] AND “treatment”[Title/Abstract]) OR (“HME”[Title/Abstract] AND “pediatric”[Title/Abstract] AND “seizures”[Title/Abstract] AND (“surgery”[Title/Abstract] OR “hemispherectomy”[Title/Abstract] OR “hemispherotomy”[Title/Abstract])) OR (“hemimegalencephaly”[Title/Abstract] AND “pediatric”[Title/Abstract] AND “seizures”[Title/Abstract]). We used an advanced search strategy with the following terms for Google Scholar: (“hemimegalencephaly” OR “HME”) AND pediatric AND (epilepsy OR seizures) AND (treatment OR surgery OR hemispherectomy OR hemispherotomy OR outcome). We used an advanced search strategy with the following terms for Scopus:

1. (“hemimegalencephaly” AND “pediatric” AND “epilepsy” AND “treatment”).
2. (“HME” AND “pediatric” AND “seizures” AND (“surgery” OR “hemispherectomy” OR “hemispherotomy”)).
3. (“hemimegalencephaly” AND “pediatric” AND “seizures”).

Variables included demographic information, medical and family history, genetics, seizure onset and semiology, localization, HME subtype, associated syndromes, neurological and imaging findings, Electroencephalogram (EEG) patterns, antiseizure medication use, and treatment response. Surgical variables included age at surgery, procedure type (e.g., functional hemispherectomy, anatomic hemispherectomy, hemispherotomy, callosotomy, lobectomy), and postoperative Engel classification.

### 2.3. Selection, Data Extraction, and Analysis

The primary reviewers (FM, MVM) screened all titles and abstracts and identified articles as being either “potentially relevant” or “irrelevant” to the research question based on the inclusion and exclusion criteria described above. Full-text copies of articles identified as potentially relevant were retrieved and individually assessed for inclusion in the review stage. Rayyan, a web platform designed to help in systematic literature reviews, was used by the reviewers and the supervisor to remove duplicates. Disagreement on inclusion or exclusion of studies was resolved by consensus achieved through the supervision process of this study on Rayyan. After reaching consensus, we collected the following information from each article: the author/year, methods, number of participants, and study design. We also extracted the main results, including each study’s outcome measures and main limitations. We analyzed the studies’ primary and secondary goals and gathered the main conclusions from each study by tabulating them into tables.

### 2.4. Quality/Risk of Bias Assessment

We used the proposed Murad method for methodological quality and synthesis of case series and case reports. This method analyzes 8 questions over 4 domains: selection, ascertainment, causality, and reporting [12]. Studies answering “yes” to ≥4/8 signaling questions were classified as low risk of bias. It is crucial to consider that 3 out of the 8 questions are only applicable for cases of adverse drug events which is the reason we considered 4 as our threshold for low risk of bias. The case series and case reports that meet criteria to be included in the study have a low risk of bias.

We used the Newcastle-Ottawa tool to perform the quality assessment in observational studies. A star system was used to perform quality assessments following the Newcastle-Ottawa Scale (NOS). A study was awarded 1 star for each numbered item within the selection and exposure categories. A maximum of 2 stars were awarded for comparability, 3 for exposure, and 4 for selection. The NOS ranges from 0 to 9 stars. We considered high-quality studies as those that achieved 7 or more stars, medium-quality studies as those with 4 to 6 stars, and poor-quality studies as those with fewer than 4 stars. The observational studies that meet criteria to be included in the study are high-quality studies.

Each study was assessed independently by the primary reviewers (FM, MVM). Discrepancies were resolved by consensus achieved through the supervision process of this study.

### 2.5. Sensitivity Analysis

To assess the robustness of the pooled findings, we conducted a post hoc sensitivity analysis by re-extracting study-level data (sample size, HME subtype, and DRE status) from the 78 included studies and recalculating key pooled estimates under four alternative exclusion scenarios: (1) removal of the single largest contributing series, (2) removal of all series with ≥8 patients, (3) restriction to single-patient case reports only, and (4) restriction to multi-patient case series only. Table 1 shows each scenario’s pooled proportions for HME subtype (isolated vs. syndromic) were recomputed from the underlying counts and compared against the base-case estimates derived from the full cohort to determine whether the direction and magnitude of the findings were sensitive to the influence of individual large series or to differential representation of case reports versus case series.

In terms of HME subtype distribution, the direction of the finding (isolated more common than syndromic) is robust across every scenario. The isolated subtype stays the majority (60–74%) regardless of which studies are excluded. The magnitude shifts by up to ~14 points depending on whether case reports or case series dominate the subset, but the qualitative conclusion does not change.

In terms of surgery, FH remains more frequent than AH in every scenario. Removing Di Rocco et al. alone (one 15-patient series) widens the FH–AH gap from 37 points to 53 points, because that one study contributes 38% of all reported AH cases. More notably, restricting to case series only narrows the gap substantially (60.0% vs. 30.7%) compared with case reports (71.4% vs. 17.1%). This suggests the reported FH predominance is inflated by the case report literature plausibly causing a publication-pattern effect (e.g., individual FH cases may be more likely to be written up than AH cases) rather than a true difference in clinical practice.

## 3. Results

We reviewed 161 reported cases including 93 males and 68 females. Table 2 shows demographics from the study. The age range is 0–18. The mean age is 3.5 years. The clinical presentation of hemimegalencephaly is most commonly with seizures, with approximately 70% (109/160, one case did not report seizures) of our sample size developing seizures before the first year of life. Focal-onset impaired awareness seizures most commonly reported in 48.7% (78/160, 1 case did not report seizures) of the cases, followed by infantile spasms and generalized tonic–clonic seizures. The characterization and quantification of seizures is difficult given the minimal detail into seizure semiology. Only 15 cases meeting criteria for inclusion in the systematic review report positive genetic information with the most commonly reported variants being PIK3CA in four cases and NPRL3 in three cases, followed by TSC1 in three cases, and RHEB, TSC2, MTOR, AKT3, and TRIO each reported once. The origin of genetic testing between brain, blood, or buccal swab has been specified for those cases with available information. Despite the lack of specific genetic reports, 59 cases report associated neurocutaneous disorders such as 18 cases of Ito’s hypomelanosis, 13 cases of linear nevus syndrome, nine cases of tuberous sclerosis, three cases of epidermal nevus syndrome other than linear nevus syndrome, three cases of Proteus syndrome, and two cases of neurofibromatosis. Additional associated epilepsy syndromes include seven cases of Ohtahara syndrome and two cases of West syndrome. Finally, associated developmental disorders include one case of trisomy 21 and one case of autosomal dominant intellectual developmental disorder-44 with microcephaly (MRD44).

Most of the reported cases were isolated HME up to 110, followed by 49 syndromic cases, and only two were total HME. Epilepsy is a common comorbidity. Drug-resistant epilepsy was present in approximately 73.2% (118/161) of the sample size. Phenobarbital is usually a first line given that seizures start in the neonatal period, but afterwards patients try multiple antiseizure medications without success which is the reason epilepsy surgery is common. Out of 161 patients, 110 (67%) had neurosurgeries. Seventy patients had a functional hemispherectomy, twenty-nine patients had an anatomical hemispherectomy, and eleven patients had other surgeries such as decortication or callosotomies. Table 3 shows the distribution of these findings. The HME type numbers correlate with the DRE yes/no variable to represent a total number of cases with DRE and HME.

In terms of diagnostic workup for the epilepsy, EEG in HME generally shows hemihypsarrhythmia, which later evolves into high-frequency background activity with almost continuous spikes, sharp waves, and spike-and-wave discharges over the malformed hemisphere that progressively involve the contralateral hemisphere [61,85]. Most of these cases end up having drug-resistant epilepsy with multiple anti-seizure medications requiring epilepsy surgery [61,85]. Some patients show asymmetric suppression-burst patterns, especially in early infancy [61].

Imaging also is part of the diagnostic workup for HME, and prenatal or postanal MRI typically shows hemispheric enlargement, often accompanied by ipsilateral ventriculomegaly, asymmetry, or dimorphism. Midline contralateral displacement and hypervascularization are also frequently observed [86,87]. Cortical abnormalities are also common, such as pachygyria or polymicrogyria, cortical thickening, gray matter heterotopia or gliosis [86,87]. The observed brain abnormalities underscore the importance of advanced prenatal imaging techniques, which has proven highly sensitive for detecting cortical abnormalities in utero if there are suspicions with prenatal ultrasounds. Imaging is also a crucial tool to decide approach to epilepsy surgery [1,35,56,63,66,67,75,82].

In terms of surgery, functional hemispherectomy (FH) (used commonly interchangeably with hemispherotomy given similar techniques) has become the most common procedure, over anatomical hemispherectomy (AH). FH was broadly comparable in terms of seizure control and long-term complications given no significant statistical difference comparted to AH [88]. However, it is important to consider the need for revision or reoperation in FH related to a less invasive procedure and sometimes incomplete disconnection [88,89]. The most common post-surgical complications included contralateral hemiparesis and hydrocephalus, while severe complications such as intracranial hemorrhage, excessive blood loss, and mortality have also been observed [88,90].

Post-surgical outcomes can be reported using the Engel classification as a standard score to understand seizure outcomes after epilepsy surgery [91]. In our review, only six studies reported Engel classifications as seen in Table 4. Nonetheless, these studies represent about 42.7% (47/110) of the sample size.

Definition of different procedures:

• Functional Hemispherectomy: Surgical disconnection of one cerebral hemisphere from the rest of the brain while leaving much of the hemisphere’s tissue in place.
• Anatomic Hemispherectomy: Complete surgical removal of one cerebral hemisphere, including the cortex, subcortical structures, and corpus callosum.
• Decortication: Surgical removal of the cerebral cortex from one hemisphere while sparing the underlying white matter and subcortical structure.

## 4. Discussion

This study provides a better understanding of hemimegalencephaly and its relationship with drug-resistant epilepsy. It is crucial to note that given the majority of studies are case reports and case studies, the reported information in most articles is biased, incomplete and not standardized, which is not a surprise, but it is necessary to mention as demonstrated in our bias assessment. From our data, we can extrapolate the information to generate awareness and promote earlier diagnosis and prompter individualized treatments.

Similar to other reported case reports and studies with large cohorts [90,92], our study identified that the isolated HME form was the most frequently reported which replicates broader and generalizable data. Most of those cases do not report genetic testing which could be a reasonable explanation to classify a case as isolated HME instead of syndrome if no neurocutaneous findings or other pathognomonic signs are noticeable on physical exams. This supports the idea of using genetic testing to inform and develop a better phenotype based on genetic variations to improve diagnostic precision and facilitate early treatment and management.

The genetic landscape of HME has shifted significantly with the discovery that it is largely a mosaicism-driven disorder. While previous understanding focused on broader developmental errors, modern genomic studies have solidified the role of the PI3K/AKT–mTOR pathway as the primary driver of this condition [7]. Additionally, the NPRL3 gene encodes for a subunit of the GATOR complex which helps regulate the mTOR signaling pathway [10]. This pathway is responsible for regulating fundamental cellular processes such as growth, proliferation, and differentiation. When a somatic mutation occurs early in neurogenesis, it leads to the characteristic uncontrolled overgrowth of a single cerebral hemisphere. These variants are typically mosaic, meaning they occur after fertilization and are only present in a subset of cells. This explains why standard blood-based genetic tests often return normal results, as the variant may be localized strictly to the affected brain tissue [7].

The inclusion of the TSC1 and TSC2 genes within this pathway provides a critical mechanistic link between HME and tuberous sclerosis complex [93,94]. These genes normally function as inhibitory “brakes” on the mTOR pathway; their loss of function leads to the same pathobiological endpoint as gain-of-function variants in genes like AKT3 or PIK3CA [93,94]. This endpoint is characterized by significant cellular hypertrophy and cortical dyslamination. Our study found positive genetic testing in only 9.3% (15/161) of the reviewed cases. Of those cases, 26.6% (4/15) were PIK3CA variants, 20% (3/15) were NPRL3 variants, 20% (3/15) were TSC1 variants and the missing 33.3% (5/15) were individual cases reporting RHEB, TSC2, MTOR, AKT3, and TRIO variants, about 6.66% (1/15) each. These results align with other studies with similar results for focal cortical malformations [95,96]. In clinical practice, identifying these variants is transformative for diagnosis, but in resource-limited settings where “deep” sequencing of brain tissue or saliva is unavailable, the clinician must pivot to a rigorous neuro-cutaneous examination to bridge the diagnostic gap [93,94].

In these environments, HME is often recognized as the neurological manifestation of a broader overgrowth syndrome through specific physical markers. For instance, the presence of hypopigmented streaks and swirls following Blaschko’s lines is a hallmark of Hypomelanosis of Ito, which often indicates chromosomal mosaicism highly associated with HME [22,34,63]. Similarly, identifying ash-leaf spots, facial angiofibromas, or Shagreen patches can immediately point toward a tuberous sclerosis diagnosis. These skin findings serve as accessible, non-invasive proxies for the underlying genetic disruptions that drive the brain’s malformation, allowing for a clinical classification even when molecular confirmation is out of reach [22,34,63].

Beyond skin-deep markers, other somatic findings provide essential prognostic value and hint at the timing of the initial genetic mutation. Macrocephaly is frequently observed at birth, reflecting the massive enlargement of the affected hemisphere. When HME is accompanied by homolateral facial hemihypertrophy or somatic hemihypertrophy, it suggests that the causal mutation occurred early enough in embryonic development to affect multiple germ layers, including both the mesoderm and ectoderm. This systemic involvement often signals a more complex clinical course, requiring the clinician to monitor for extracranial complications such as renal or cardiac issues depending on the suspected syndrome [22,34,63].

Clinical phenotyping in HME is not merely a tool for classification but a vital component of the management roadmap. Distinguishing between isolated HME and syndrome-associated HME dictates the urgency of various interventions. While isolated HME might focus primarily on the management of refractory epilepsy and potential surgical evaluation, syndrome-associated cases require a multidisciplinary approach to screen for systemic tumors or vascular malformations. By integrating these physical findings with the known molecular pathways, healthcare providers can better determine a patient’s prognosis and tailor their surveillance strategies to the specific risks associated with the underlying mosaic disorder.

Besides the clinical diversity and complexity of these cases, these patients present with seizures, most commonly focal-onset in nature given the unilateral lesion to the brain with correlates with our study’s data [61,85]. Additionally, infantile spasms are commonly associated as well given mutations in the mTOR pathway causing an abnormal activation, correlated in animal models with focal seizures, and an increased propensity for spasms, demonstrating the direct mechanistic connection probably in the setting of neuronal immaturity, cortical dysplasia, GABAergic pathways dysfunction, and chloride homeostasis dysregulation [97].

Epilepsy surgery is one of the most common approaches to DRE in cases with focal etiologies for epilepsy [88]. Our sample includes about 70% (110/161) of patients who had epilepsy surgery and 42.7% (47/110) of those cases report an Engel classification. Of this sample, more than 51.1% (24/47) of the patients were completely seizure free. Despite the small sample size, these results correlate with other studies with larger cohorts reporting excellent outcomes with the majority of patients achieving a Ia-Ic score with significant improvement in epilepsy control [51,86,90,93].

Despite the benefits from surgery, not every child is a candidate which has spurred interest in minimally invasive alternatives such as staged transarterial embolization for infants too unstable for open surgery, with early single-center data showing comparable seizure and developmental outcomes, though long-term safety and efficacy remain unestablished [9,98]. Also, the discovery that HME arises from somatic mosaic PI3K–AKT–mTOR pathway variants has opened the possibility of targeted medical therapy as an adjunct or alternative to surgery, but whether molecular diagnosis should redirect management remains actively debated rather than standard practice [93].

Other associated aspects before and after epilepsy surgery like developmental, language, and motor delay, intellectual disability, and psychosocial skills are generally not reported but should be managed in a multidisciplinary fashion with physical therapy, occupational therapy, early intervention programs, speech therapists, and neuropsychologists depending on the severity and individual needs of the case [99,100].

We propose a clinical framework (Figure 2) based on the presented information to guide the approach to HME cases. It should be emphasized that the transition from failure of antiseizure medications to hemispherectomy should also reflect the need for comprehensive multidisciplinary presurgical evaluation and individualized selection of surgical technique.

### Limitations

One of the biggest limitations is the lack of long-term neurological follow-up in the existing literature. This gap hindered the ability to evaluate prolonged post-treatment outcomes. Additionally, the constantly changing and evolving field complicates the standardization of seizure semiology and DRE definition, especially when using primarily case reports and case studies to gather the data used in the manuscript as well as the comparability of surgical outcomes without a unique score such as Engel’s classification. Data about surgical outcomes and genetic testing is only reported in certain cases which is impactful for the final conclusion of the study. There is also a possibility of patient overlap given the rarity of this syndrome. However, this has been minimized by the strategies mentioned in the Section 2. Finally, other limitations are that we only included English-language studies and protocol nonregistration.

The data has been compared with other studies and reflect similar results despite the small sample with these specific data.

## 5. Conclusions

Hemimegalencephaly is a disease with a broad clinical and genetical spectrum. The findings in this study aid in the understanding of its diagnosis and outlines the spectrum of the known disease phenotype. Published HME cases frequently involve early-onset epilepsy. Hemispheric surgery can provide substantial seizure reduction in selected patients, despite the risk of postoperative complications. Early diagnosis and coordinated multidisciplinary care are essential for achieving the best clinical outcomes in patients with HME. Broader studies including possible milder phenotypes and long-term follow-up are needed to enhance understanding and refine management strategies of this disease. Future research must prioritize genetic testing, standardization of seizure semiology, and post-surgical outcomes to determine whether surgical or medical treatments offer lasting benefits for HME patients.

## Figures and Tables

**Figure 1:**
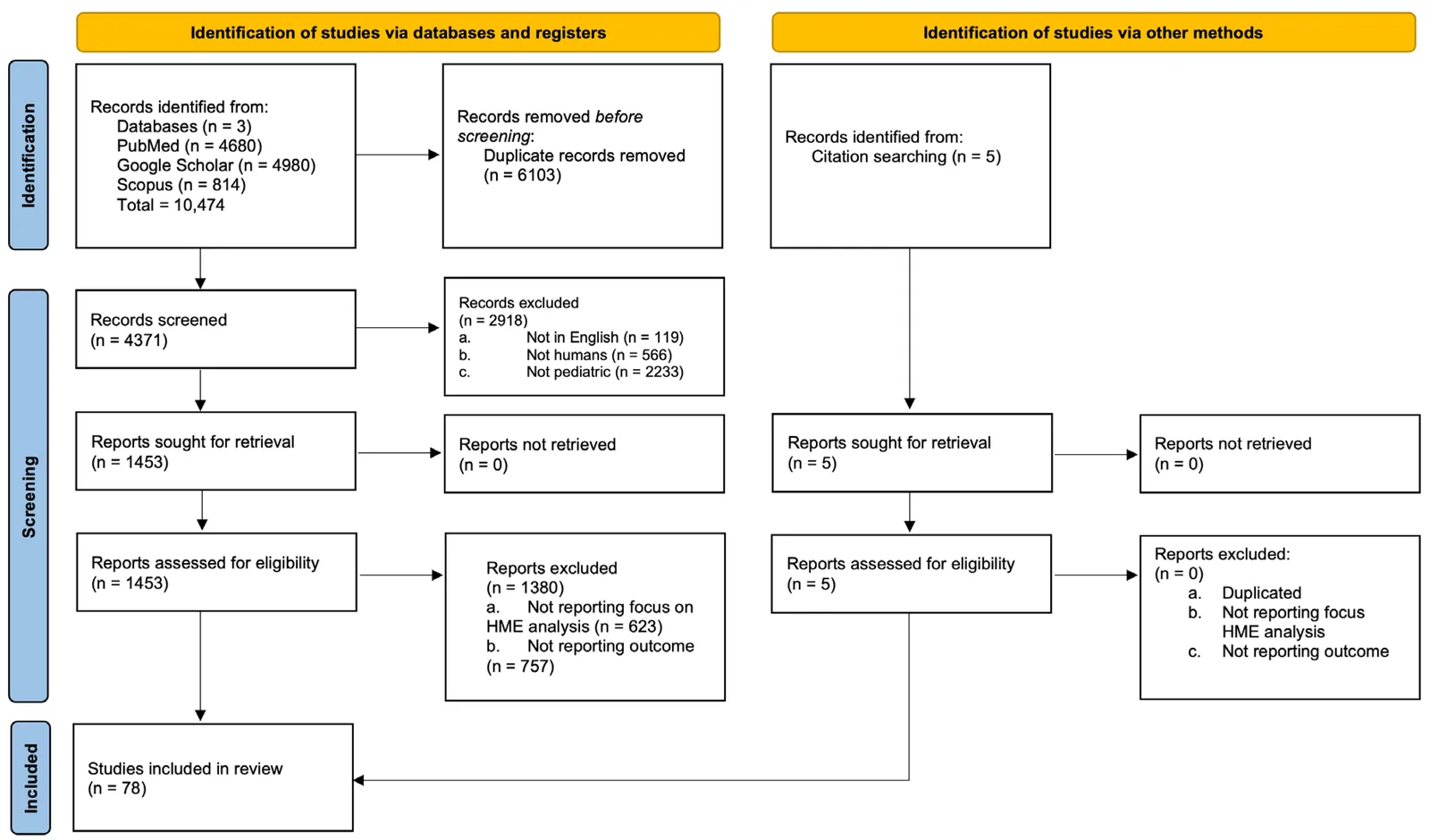
PRISMA.

**Figure 2:**
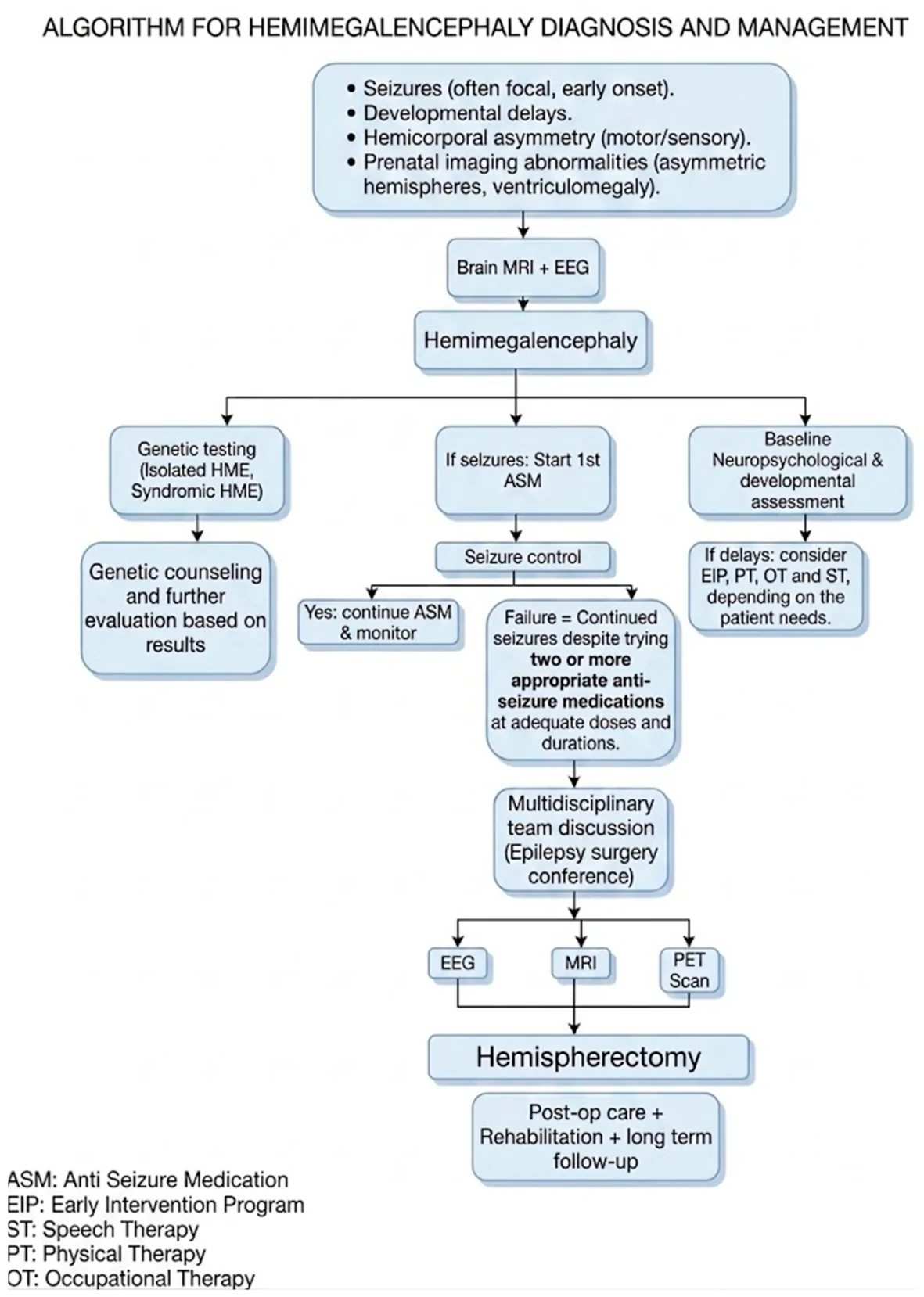
Proposed clinical framework for hemimegalencephaly.

**Table 1:** Sensitivity analysis.

Scenario	Studies	Patients (HME-Type Denom.)	Isolated	Syndromic	Total-Type	FH	AH
Base case (all studies)	78	161	68.30%	30.40%	1.20%	63.60%	26.40%
Excl. Di Rocco et al. (largest AH contributor)	77	146	69.20%	29.50%	1.40%	71.60%	18.90%
Excl. 6 largest series (*n* ≥ 8 each)	72	93	62.40%	35.50%	2.20%	71.40%	17.90%
Case reports only (*n* = 1)	61	62	59.70%	37.10%	3.20%	71.40%	17.10%
Case series only (*n* > 1)	17	99	73.70%	26.30%	0.00%	60.00%	30.70%

**Table 2:** Study demographics.

Author	Year	Country	Mean Age (y)	Sample Size	Sex	Associated Disorder	Seizure Type	Genetic Variants/Test/Pathogenicity
Ai et al. [13]	2015	Taiwan	1	1	1F		Right sided focal seizures	
Alexopoulos et al. [14]	2005	USA	0.6	3	2F, 1M	Epidermal nevus syndrome	Focal motor seizures, GTC	
Alfonso et al. [15]	2004	USA	0	1	1M		Nonspecific seizures, status epilepticus	
Alfonso et al. [16]	1998	USA	0.5	2	1F, 1M		GTC. Limb jerk with apnea cyanosis and eye blinking.	
Alvarez et al. [17]	2011	Spain	3	1	1F		Seizures non specified	
Agrawal et al. [18]	2017	India	0	1	1F		No seizures	
Bastos et al. [19]	2007	Brazil	0	2	2F	Proteus syndrome	Epileptic spasms	
Becherini et al. [20]	2008	Italy	8	1	1F		Hypomotor seizures, asymmetric spasms, hypermotor seizures in clusters, status epilepticus	
Broumandi et al. [21]	2004	USA	0	1	1M		Apnea spells	
Calzolari et al. [22]	1996	Italy	0	1	1M		GTC	
Carozza et al. [23]	2023	USA	0	1	1M		Right sided clonic movements	
Chand et al. [24]	2018	Pakistan	1	1	1F		Jerky movements, focal seizures	-
Chandrasekar et al. [10]	2021	USA	0	1	1F		Focal motor seizures, GTC, sublinical seizures	NPRL3 (Rapid WGS from blood) chr16:161898-164745x1
Chapman et al. [25]	2008	USA	5	1	1F	Hypomelanosis of Ito	Focal impaired awareness seizures + atonic seizures	
Chrastina et al. [26]	2015	Czech Republic	18	1	1M		Myoclonic seizures, focal motor seizures, GTC	
Cornelius et al. [27]	2017	India	6	1	1M	Linear nevus syndrome	Focal motor seizures, epilepsis partialis continua	
Cuddapah et al. [28]	2015	USA	5	1	1M	TSC	Brief bilateral flexion of extremities, epileptic spasms, nonconvulsive status epilepticus	TSC1 (C > T at nucleotide 2074, pathogenic)
Cusmai et al. [29]	1990	Italy	7	2	2M	Neurofibromatosis	Left clonic jerks and asymmetrical spasms	
Di Rocco et al. [2]	2002	Italy	2.2	15	11M, 4F	1 Hypomelanosis of Ito	Seizures nonspecified	
D Shields et al. [30]	1999	USA	4	1	1F		GTC, infantile spasms	
Edmonds et al. [5]	2024	USA	0.4	9	5M, 4F		Focal motor, nonspecific seizures	4: PIK3CA (3 brain, 1 buccal swab, mosaicism ×4, c.1633G > A, c.1624G > A, c.3140A > G, c.1636C > A), 2: NPRL3 (blood, germline 2x, 1. Deletion chr16:161898-1647451x1 (3 kb)), 2. c.274C > T) 1: MTOR (brain, mosaicism, c.1633G > A), 1: AKT3 (brain, mosaicism, c.49G > A), 1: unreported
Elting et al. [31]	2009	The Netherlands	0	1	1M		Myoclonic jerking, tonic extension of the limbs, status epilepticus	
Galluzzi et al. [32]	2002	Italy	0	1	1F		Clonic bilateral	
Ganguly et al. [33]	2018	India	17	1	1M		GTC, myoclonic jerks, hemimyoclonus	
Gökçe et al. [34]	2022	Turkey	5.5	2	1M, 1F	West syndrome, Hypomelanosis of Ito	GTC	
Golhar et al. [35]	2016	India	0	1	1M		Multifocal clonic convulsions, infantile spasms	
Gowda et al. [36]	2015	India	0	1	1F	Ohtahara syndrome	Tonic neck flexor spasms	
Griffiths et al. [37]	1998	UK	3.5	1	1F	TSC	Seizures nonspecified	
Guerra et al. [38]	2007	Italy	2	1	1M	TSC	Tonic seizures, myoclonic jerks	TSC2 (mutation in exon 37)
Gunbey et al. [39]	2025	Turkey	0.3	14	7M, 7F		Focal motor clonic, focal motor tonic	
Guzzetta et al. [40]	2002	Italy	0	1	1M	Ohtahara syndrome	Tonic spasms, atonias, focal seizures	
Hee Kim et al. [41]	2016	USA	6	1	1F		Eye fluttering and hemiconvulsion	
Higurashi et al. [42]	2011	Japan	2	1	1M		Motion arrest, apnea, facial cyanosis, epileptic spasms in clusters,	
Honda et al. [43]	2013	Japan	6.4	12	6M, 6F	Hypomelanosis of Ito, linear nevus sebaceus, facial lipomatosis	GTC, spasms	
Humbertclaude et al. [44]	1998	France	4	1	1M		Clonic seizures, status epilepticus, infantile spasms, GTC	
Jahan et al. [45]	1997	USA	0	1	1F		GTC	
Jaiswal et al. [4]	2021	USA	1	1	1M		Infantile spasms, GTC	
Kakish et al. [46]	2022	USA	1	1	1M		Infantile spasms	
Kentab et al. [47]	2017	Saudi Arabia	10	1	1M	Hypomelanosis of Ito	Focal motor seizures, GTC	
Kometani et al. [48]	2009	Japan	1	1	1M		Tonic seizures, apneic and hemitonic seizures, asymmetrical epileptic spasms	
Konkol et al. [49]	1990	USA	5	2	2F		Focal motor seizures, myoclonic seizures, infantile spasms, GTC	
Kumar et al. [50]	2023	India	18	1	1F		Left-sided focal seizures	
Lang et al. [51]	2014	USA	0	1	1M		Infantile spasms, simple focal seizures, tonic seizures progressing to status epilepticus	
Lettori et al. [52]	2007	Italy	7.7	10	6M, 4F		Focal seizures, spasms, GTC	
Maher et al. [53]	2003	USA	5	1	1M		Tonic posturing, nystagmoid eye movement, apnea, partial seizures	
Makridis et al. [54]	2021	Germany	0	1	1M		Apnea, clonic seizures	
Martinez et al. [55]	1992	Madrid	4	1	1F	Ohtahara syndrome	GTC, hemiclonic, tonic spasms	
Mathis et al. [56]	1995	USA	1	1	1F		Focal seizures	
Mohamed et al. [57]	2022	Qatar	1	1	1M		Clonic seizures, myoclonic jerks, status epilepticus, GTC	
Nagahama et al. [58]	2017	USA	3	1	1M		Focal seizures	
Nakashima et al. [59]	2012	Japan	2	1	1M		Tonic seizures, GTC, focal myoclonus	
Ohta et al. [60]	1994	Japan	9	1	1F		Infantile spasms, lennox–gastaut, GTC	
Ohtsuka et al. [61]	1998	Japan	14	1	1F	Ohtahara syndrome	Spasms, GTC, generalized atonic seizures, myoclonic	
Okanari et al. [62]	2014	Japan	3	1	1M	T21, Hypomelanosis of Ito	GTC	
Olmos-López et al. [63]	2016	Mexico	9	2	2F	West syndrome	Infantile spasms, GTC	
Pavičić Klancir et al. [64]	2023	Croatia	4	1	1F	Ohtahara syndrome	Unilateral right extensor tonic spasms + focal motor seizures + hemiconvulsions with generalization	
Pelayo et al. [65]	1994	USA	3	1	1M		Head drops and myoclonic jerks	
Pepi et al. [3]	2022	Italy	9.2	4	2M, 2F		Assymetric spasms, tonic seizures	
Pepper et al. [66]	2022	UK	5	5	3F, 2M	TSC	Focal, GTC, eyelid flickering	
Raus et al. [67]	2016	Romania	10	1	1M		Infantile spasms, focal seizures	
Rintahaka et al. [68]	1992	USA	2.3	8	8M		GTC, myoclonic jerks	
Rondagh et al. [69]	2025	Netherlands	0	1	1F	TSC	Clonic jerking	TSC1 (blood, somatic, pathogenic, Chr9; GRCh37)
Roy et al. [70]	2017	India	0	1	1F	Ohtahara syndrome	Focal motor seizures	
Roza et al. [71]	2022	Romania	13	1	1F	MRD44	Atonic seizures, generalized tonic–clonic	TRIO (WES, variant of unknown significance, missense variant c.2335A > G p.(Lys779Glu), exon 13)
Sakuma et al. [72]	2005	Japan	5	1	1M	TSC	Focal motor seizures, tonic seizures	
Sakuta et al. [73]	1991	Japan	5	1	1M		Infantile epileptic encephalopaty, infantile spasms, GTC	
Salinas et al. [74]	2018	Argentina	1	1	1F		Clonic seizures	RHEB (HDES, brain, likely pathogenic, NM_005614:c.119A > T: p.Glu40Val)
Santana-Ramirez et al. [75]	2014	Mexico	0	1	1M		Partial seizures with right focal and multifocal tonic–clonic limb movements	
Sarnat et al. [76]	2012	Canada	3.6	3	3M	Ohtahara syndrome, Proteus syndrome	Focal seizures, spasms	
SS Ajaj et al. [77]	2022	Libya	0	1	1M		Neonatal convulsions	
Serletis et al. [78]	2022	USA	0	1	1F	TSC	Jerking in the right arm and leg	TSC1 (buccal, pathogenic, heterozygous c.2041 + 1G > A mutation in intron 16)
Shilpa et al. [79]	2015	India	3	1	1F		Multifocal clonic seizures, infantile spasms	
Shim et al. [1]	2022	South Korea	1	1	1F	TSC	Clonic focal seizures	
Shiroishi et al. [80]	2010	USA	0	1	1F		Not specified if seizures or not	
Taha et al. [81]	1994	USA	4.2	5	3M, 2F		Focal motor seizures, GTC	
Wintermark et al. [82]	2009	Switzerland	0	1	1M		Tonic seizures	
Wolpert et al. [83]	1994	USA	0	1	1M		Forced eye deviation associtted with clonic movements of the right side + clonic movement of limbs	
Yoshioka et al. [84]	1998	Japan	4	1	1F		Focal motor seizures, GTC, status epilepticus	

GTC: generalized tonic–clonic. WGS: whole-genome sequencing. WES: whole-exome sequencing. HDES: high-depth exome sequencing.

**Table 3:** HME characteristics.

Studies	HME Type			DRE	Surgery Type		
	Isolated	Syndromic	Total		Functional Hemispherectomy	Anatomical Hemispherectomy	Others
Ai et al.[13]		1		no			
Alexopoulos et al. [14]	2	1		yes	1	2	
Alfonso et al. [15]		1		yes	1		
Alfonso et al. [16]	2			yes			
Alvarez et al. [17]	1			yes	1		
Agrawal et al. [18]	1			no			
Bastos et al. [19]		2		yes			
Becherini et al. [20]	1			yes	1		
Broumandi et al. [21]	1			yes	1		
Calzolari et al. [22]			1	yes	1		
Carozza et al. [23]	1			yes		1	
Chand et al. [24]	1			yes	1		
Chandrasekar et al. [10]	1			yes	1		
Chapman et al. [25]		1		yes			
Chrastina et al. [26]	1			yes			
Cornelius et al. [27]		1		yes			
Cuddapah et al. [28]		1		yes	1		
Cusmai et al. [29]		2		no			
Di Rocco et al. [2]	9	6		yes	2	11	2
D Shields et al. [30]	1			yes	1		
Edmonds et al. [5]	6	3		-	9		
Elting et al. [31]	1			yes			
Galluzzi et al. [32]		1		-			
Ganguly et al. [33]		1		yes			
Gökçe et al. [34]		2		-			
Golhar et al. [35]	1			yes			
Gowda et al. [36]		1		-			
Griffiths et al. [37]		1		yes	1		
Guerra et al. [38]		1		yes	1		
Gunbey et al. [39]	11	3		-			
Guzzetta et al. [40]	1			yes			
Hee Kim et al. [41]	1			yes		1	
Higurashi et al. [42]	1			yes	1		
Honda et al. [43]	8	4		-	12		
Humbertclaude et al. [44]	1			yes	1		
Jahan et al. [45]	1			yes		1	
Jaiswal et al. [4]	1			no			
Kakish et al. [46]	1			yes	1		
Kentab et al. [47]		1		no			
Kometani et al. [48]	1			-	1		
Konkol et al. [49]	2			yes			
Kumar et al. [50]		1		yes			
Lang et al. [51]	1			yes		1	
Lettori et al. [52]	10			yes	2	6	2
Maher et al. [53]		1		-			1
Makridis et al. [54]	1			yes	1		
Martinez et al. [55]	1			yes			1
Mathis et al. [56]	1			yes			1
Mohamed et al. [57]	1			yes			
Nagahama et al. [58]	1			yes	1		
Nakashima et al. [59]	1			yes	1		1
Ohta et al. [60]	1			no			
Ohtsuka et al. [61]	1			yes			
Okanari et al. [62]		1		yes	1		
Olmos-López et al. [63]	1	1		yes			
Pavičić Klancir et al. [64]		1		yes	1		
Pelayo et al. [65]		1		yes			
Pepi et al. [3]	4			yes	4		
Pepper et al. [66]	4	1		yes	5		
Raus et al. [67]	1			yes			
Rintahaka et al. [68]	8			yes	5	2	1
Rondagh et al. [69]	1			yes	1		
Roy et al. [70]	1			yes	1		
Roza et al. [71]		1		no			
Sakuma et al. [72]		1		yes			
Sakuta et al. [73]		1		yes			
Salinas et al. [74]	1			yes		1	
Santana-Ramirez et al. [75]		1		no			
Sarnat et al. [76]	2	1		yes	3		2
SS Ajaj et al. [77]		1		yes			
Serletis et al. [78]		1		yes		1	
Shilpa et al. [79]	1			yes	1		
Shim et al. [1]		1		yes	1		
Shiroishi et al. [80]	1			-			
Taha et al. [81]	5			yes	1	2	
Wintermark et al. [82]			1	-			
Wolpert et al. [83]	1			yes	1		
Yoshioka et al. [84]	1			yes	1		

**Table 4:** Engel classification for post-surgical outcomes.

Studies	Surgery/Cases	Engel	Mean Follow-Up
Di Rocco et al. [2]	13 AH, 2 FH	6 Ia, 2 Ib, 2 Ic, 2 IIa, 2 IIb, 1 IVb	5.5 years
Honda et al. [43]	12 FH	6 Ia, 1 Ib, 1 Ic, 1III, 3 IV	6.5 years
Lettori et al. [52]	6 FH, 2 AH, 2 HD	4 Ia, 1 Ic, 2 IIa, 3 IIIa	6.6 years
Maher et al. [53]	1 cortical resection	Ia	4.6 years
Pepi et al. [3]	4 FH	4 Ia	6.5 years
Pepper et al. [66]	5 FH	3 Ia, 1 Ic, 1 IVb	4 years

Class I—(a) Free of disabling seizures (completely seizure free); (b) non-disabling, simple partial seizures only; (c) some disabling seizures, but free of disabling seizures for at least 2 years; (d) generalized convulsion with antiepileptic drug withdrawal only. Class II—(a) Rare disabling seizures (initially free of disabling seizures, but rare seizures now); (b) rare disabling seizures since surgery; (c) more than rare disabling seizures, but rare seizures for at least 2 years; (d) nocturnal seizures only. Class III—(a) Worthwhile improvement (worthwhile seizure reduction); (b) prolonged seizure-free intervals amounting to more than half the follow-up period, but not less than 2 years. Class IV—(a) No worthwhile improvement (significant seizure reduction); (b) no appreciable change; (c) seizures worse.

## Data Availability

Data is available upon request.

## Supplementary Materials

The following supporting information can be downloaded at: https://www.mdpi.com/article/10.3390/children13101292/s1, Table S1: PRISMA 2020 checklist.
